# Supplementary material for: From Solid-State Cluster Compounds to Functional PMMA-Based Composites with UV and NIR Blocking Properties, and Tuned Hues
Source: Nanomaterials (Basel). 2022 Dec 28;13(1):144. doi: 10.3390/nano13010144 (PMC9824331; doi:10.3390/nano13010144)
Supplement: Supplementary file 1 [file nanomaterials-13-00144-s001.zip › nanomaterials-2089460-supplementary.pdf]

Electronic Suplemmentary Information

Figure S1. ^{1}H-NMR ($CDCl_3$) of Ta_x@PMMA copolymer pellets in the oxidised form……………………………2

Figure S2. ^{1}H-NMR spectra (CD_2Cl_2) of DMAP (top) and $(TBA)_2[\{Ta_6Br^i_{12}\}Br^a_6]$ with two equivalents of DMAP (bottom)…….…2

Figure S3. Mid-FTIR spectra of DMAP (straight) and $(TBA)_2[\{Ta_6Br^i_{12}\}Br^a_6]$ with two equivalents of DMAP (dash)……3

Figure S4. DSC thermograms of Ta_1@PMMA. (straight) oxidized state heating cycle and (dot) cooling cycle and (dash) reduced state heating cycle and (dash dot) cooling cycle…………………………………….……3

Figure S5. DSC thermograms of Ta_2@PMMA. (straight) oxidized state heating cycle and (dot) cooling cycle and (dash) reduced state heating cycle and (dash dot) cooling cycle…………………………………….…4

Figure S6. DSC thermograms of Ta_3@PMMA. (straight) oxidized state heating cycle and (dot) cooling cycle and (dash) reduced state heating cycle and (dash dot) cooling cycle…………………………………….……4

Figure S7. Mid-FTIR a) Ta_1@PMMA$_{brown}$ (bottom), Ta_1@PMMA$_{green}$ (top), b) Ta_2@PMMA$_{brown}$ (bottom), Ta_2@PMMA$_{green}$ (top), c) Ta_3@PMMA$_{brown}$ (bottom), Ta_3@PMMA$_{green}$ (top)……………….5

Figure S8. Far IR spectra of a) PMMA, b) $TBA_2Ta_6Br_{18}$, c) Ta_1@PMMA$_{brown}$ (bottom), Ta_2@PMMA$_{brown}$ (medium), Ta_3@PMMA$_{brown}$ (top), d) Ta_1@PMMA$_{green}$ (bottom), Ta_2@PMMA$_{green}$ (medium), Ta_3@PMMA$_{green}$ (top)……………………………………………………………………..6

Table S1. Main far IR absorption bands for $[\{Ta_6Br^i_{12}\}Br^a_6]^{2-}$, PMMA and Ta_x@PMMA composites…….7

Figure S9. CIE chromaticity coordinates of Ta_x@PMMA (VEC = 14 in the oxidized form and VEC = 16 in the reduced form)……….7

Table S2. x, y, z and L*, a*, b colour coordinates of Ta_x@PMMA composites…………………………………..7

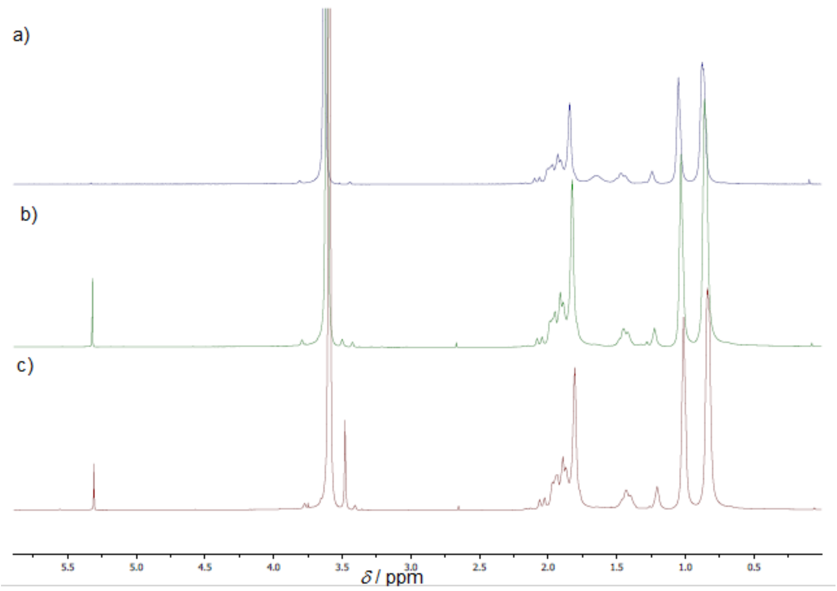

Figure S1. ^{1}H-NMR ($CDCl_3$) of Ta_x@PMMA$_{brown}$ copolymer pellets in the oxidised form: a) 1 wt%, b) 2 wt% and c) 3 wt%

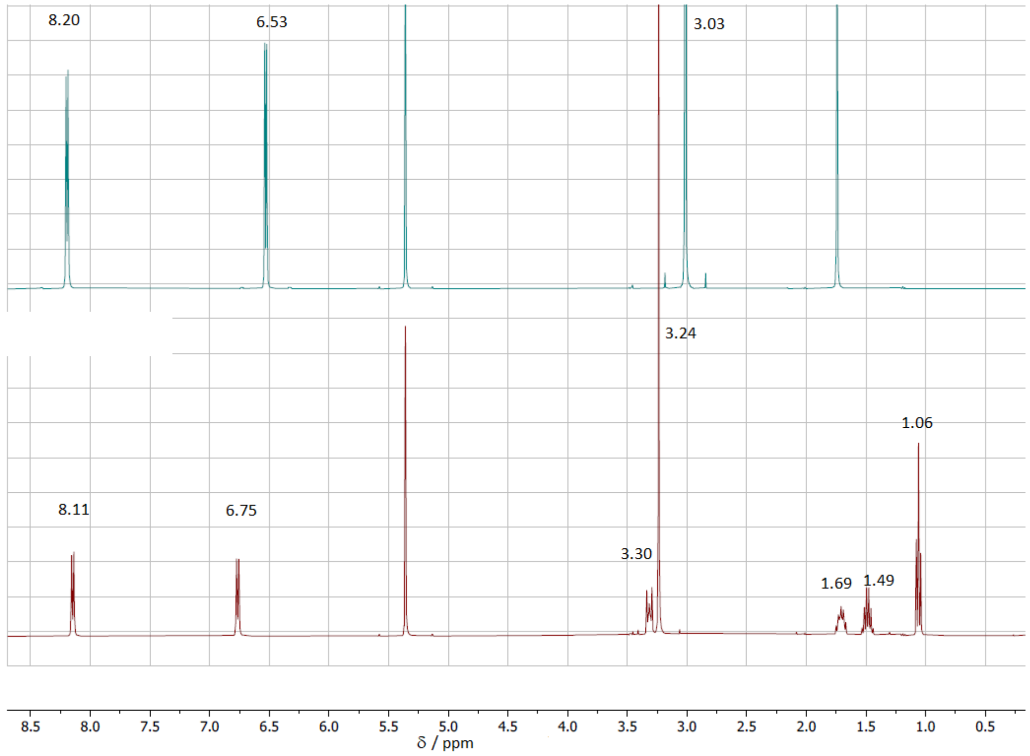

Figure S2. ^{1}H-NMR spectra (CD_2Cl_2) of DMAP (top) and $(TBA)_2[\{Ta_6Br^{i}_{12}\}Br^{a}_6]$ with two equivalents of DMAP (bottom).

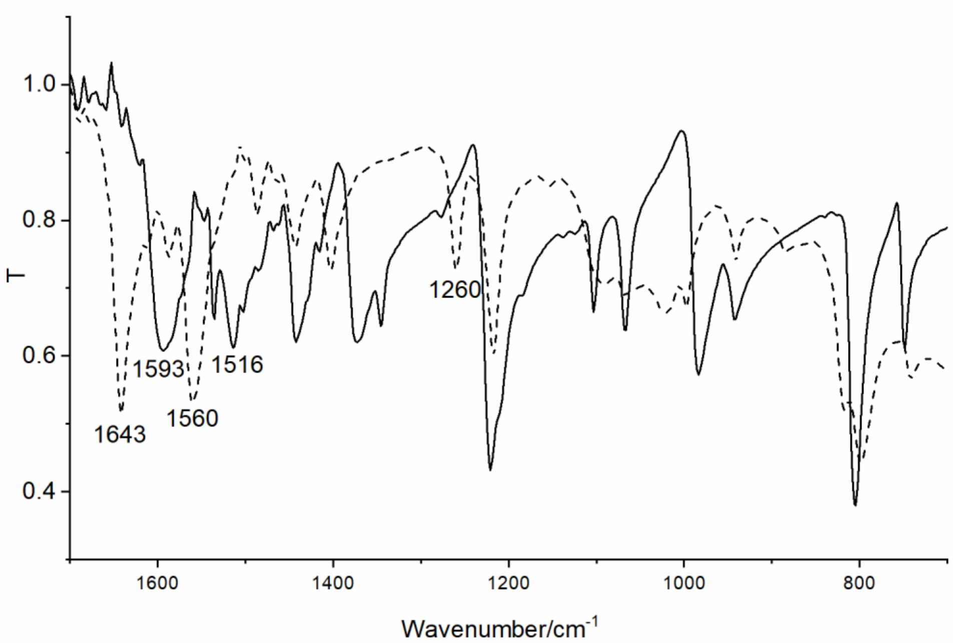

Figure S3. Mid-FTIR spectra of DMAP (solid) and $(TBA)_2[\{Ta_6Br^i_{12}\}Br^a_6]$ with two equivalents of DMAP (dash).

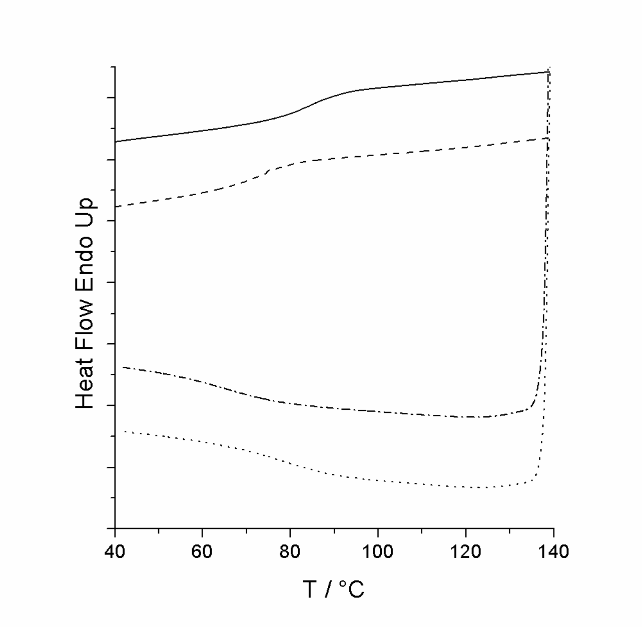

Figure S4. DSC thermograms of Ta_1@PMMA. (solid) oxidized state heating cycle and (dot) cooling cycle and (dash) reduced state heating cycle and (dash dot) cooling cycle.

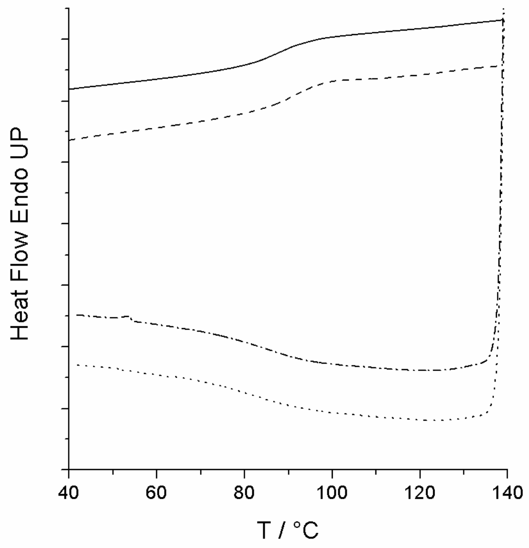

Figure S5. DSC thermograms of Ta_2@PMMA. (solid) oxidized state heating cycle and (dot) cooling cycle and (dash) reduced state heating cycle and (dash dot) cooling cycle.

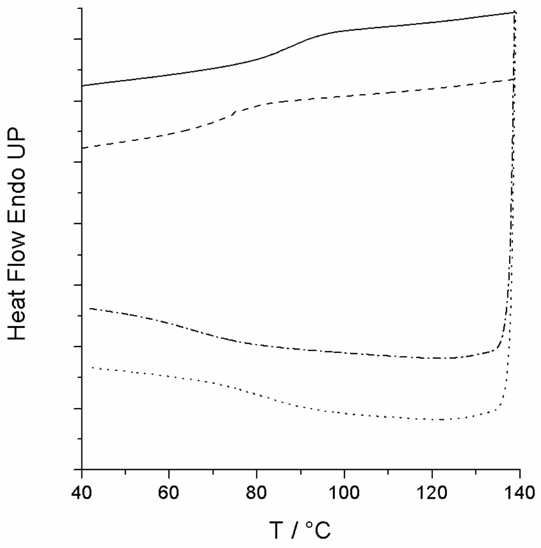

Figure S6. DSC thermograms of Ta_3@PMMA. (solid) oxidized state heating cycle and (dot) cooling cycle and (dash) reduced state heating cycle and (dash dot) cooling cycle.

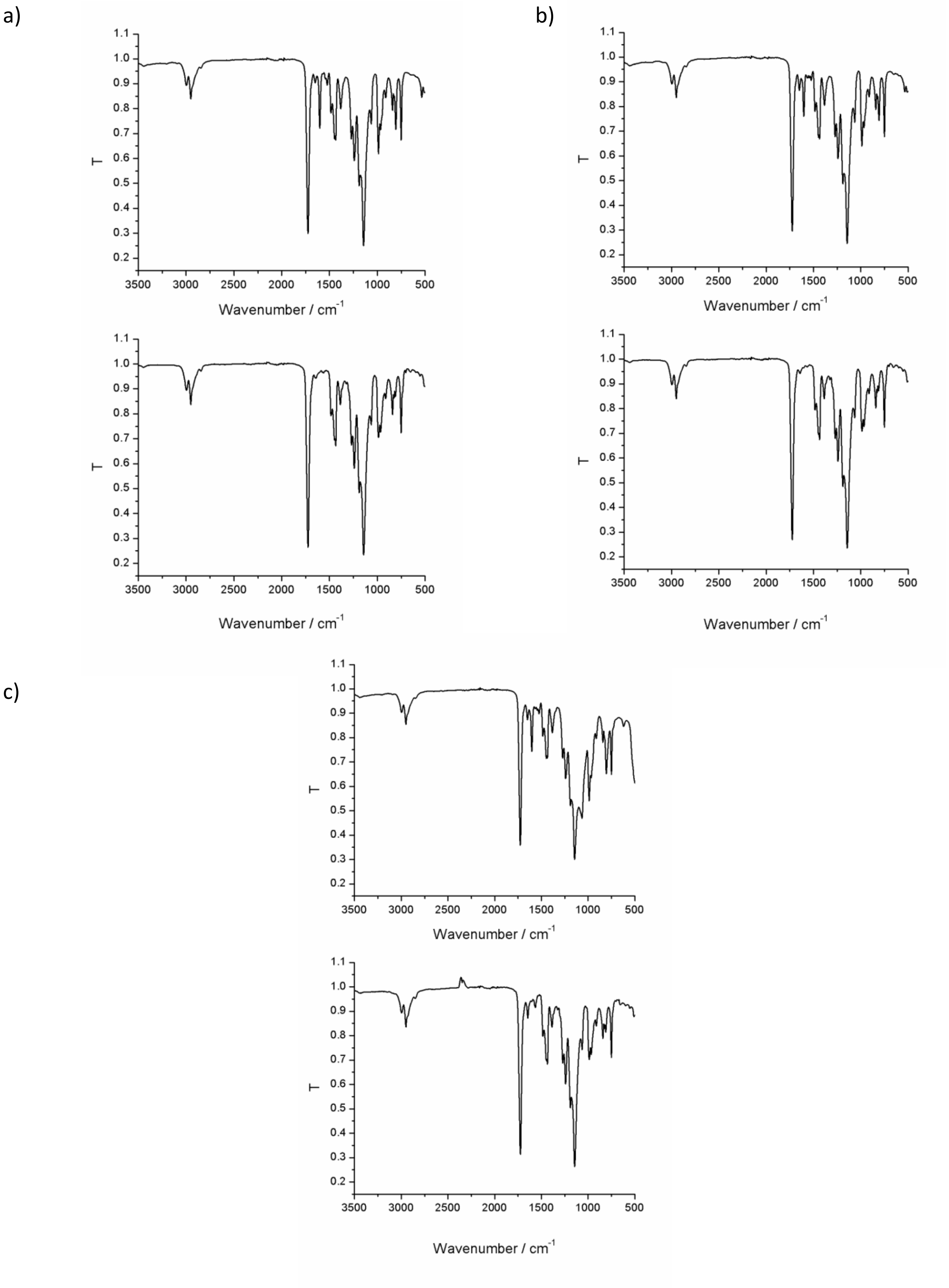

Figure S7. Mid-FTIR a) Ta_1@PMMA$_{brown}$ (bottom), Ta_1@PMMA$_{green}$ (top), b) Ta_2@PMMA$_{brown}$ (bottom), Ta_2@PMMA$_{green}$ (top), c) Ta_3@PMMA$_{brown}$ (bottom), Ta_3@PMMA$_{green}$ (top).

a)

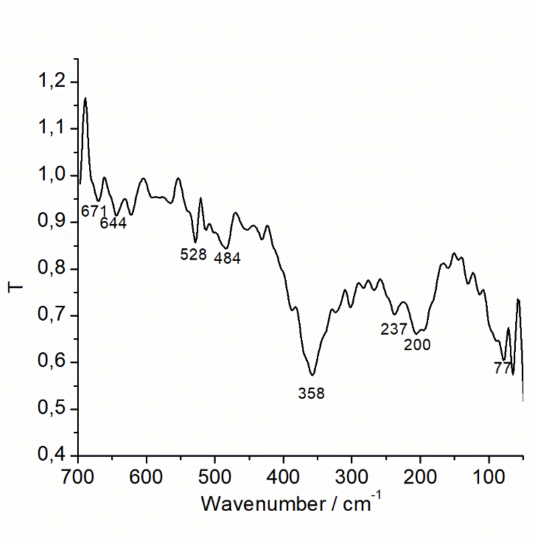

1,2
1,1
1,0
0,9
0,8
0,7
0,6
0,5
0,4
T
671
644
528
484
358
237
200
77
700
600
500
400
300
200
100
Wavenumber / cm-1

b)

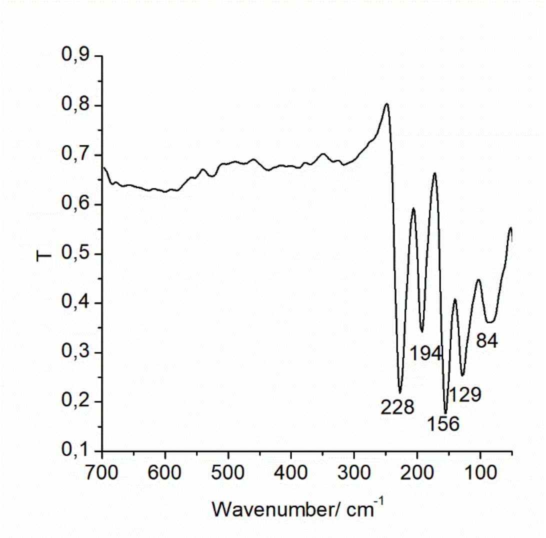

0,9
0,8
0,7
0,6
0,5
0,4
0,3
0,2
0,1
T
228
194
156
129
84
700
600
500
400
300
200
100
Wavenumber/ cm-1

c)

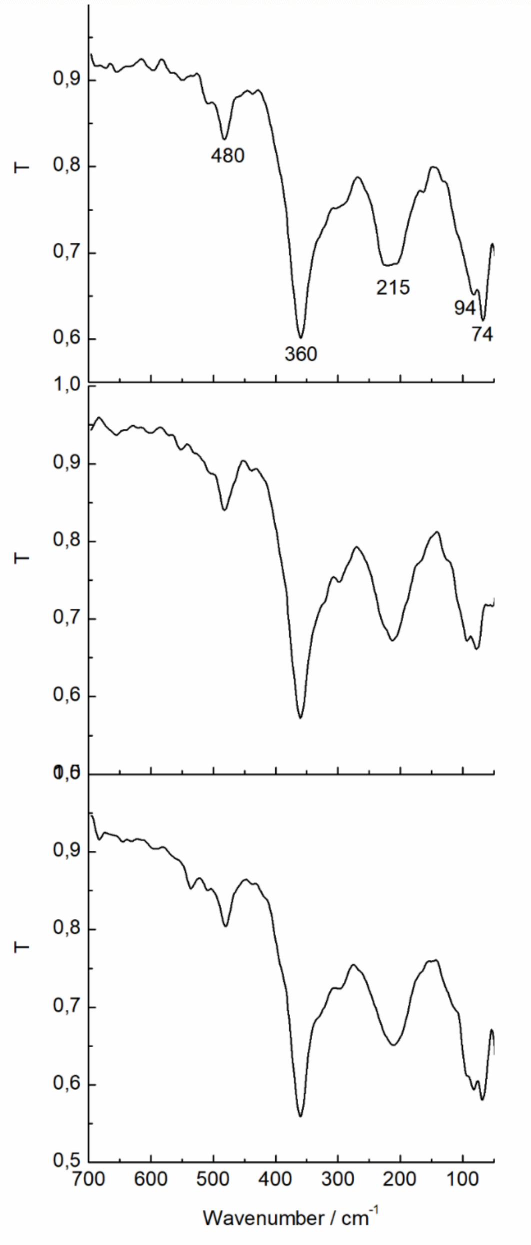

T
480
360
215
94
74
1,0
0,9
0,8
0,7
0,6
0,5
700
600
500
400
300
200
100
Wavenumber / cm-1

d)

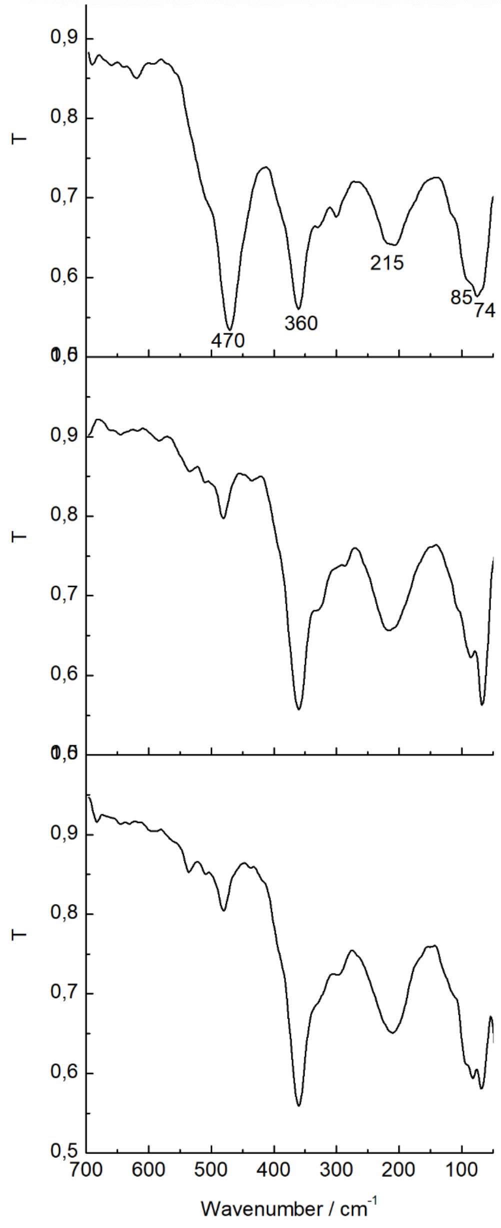

T
470
360
215
85
74
0,9
0,8
0,7
0,6
0,5
700
600
500
400
300
200
100
Wavenumber / cm-1

Figure S8. Far IR spectra of a) PMMA, b) $TBA_2Ta_6Br_{18}$, c) Ta_1@$PMMA_{brown}$ (bottom), Ta_2@$PMMA_{brown}$ (medium), Ta_3@$PMMA_{brown}$ (top), d) Ta_1@$PMMA_{green}$ (bottom), Ta_2@$PMMA_{green}$ (medium), Ta_3@$PMMA_{green}$ (top).

Table S1. Main far IR absorption bands for $[\{Ta_6Br^i_{12}\}Br^a_6]^{2-}$, PMMA and Ta_2@PMMA composites

Reported for $(TEA)_2[\{Ta_6Br^i_{12}\}Br^a_6]$[1]	Found for $(TBA)_2[\{Ta_6Br^i_{12}\}Br^a_6]$	Reported for PMMA	Found for PMMA	Ta-2@PMMAox	Ta -2@PMMA-red
-----	-----	483 w	484 w	482 w	480 w
-----	-----	365 br,vs	360 br, vs	360 br, vs	360 br, vs
230 vs	228 vs	228 vbr	237 w	227-200 vbr, m	221-200, vbr, m
196 vs	194 vs	----	200 vbr, m		
161 vs	156 vs	----	----	----	----
134 s	129 s	----	----	----	----
98 m	---	----	----		
82 m	84 br, m	----	94 -75 br, m, sp	90 – 75 br, m, sp	95 – 75, br, m, sp

vs: very strong; s: strong; m: medium; w: weak; br: broad; vbr: very broad; sp: splitted

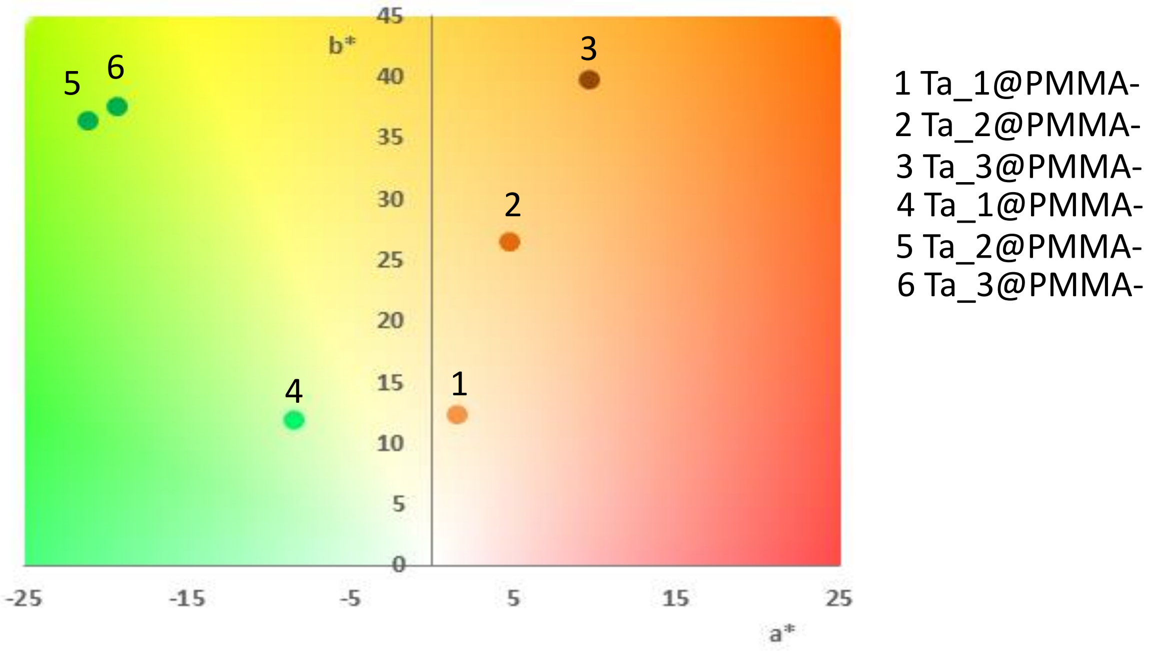

Figure S9. CIE chromaticity coordinates of Ta_x@PMMA (VEC = 14 in the oxidized form and VEC = 16 in the reduced form)

Table S2. L*, a*, b color coordinates of Ta_x@PMMA composites

sample	*L**	*a**	*b**	Color
Ta-1@$PMMA_{brown}$	92.2	1.49	12.38	
Ta-2@$PMMA_{brown}$	84.64	4.71	26.45	
Ta-3@$PMMA_{brown}$	79.18	9.52	39.91	
Ta-1@$PMMA_{green}$	92.14	-8.53	11.87	
Ta-2@$PMMA_{green}$	76.47	-21.12	36.36	
Ta-3@$PMMA_{green}$	71.75	-19.33	37.53	
